# Plants traditionally used to make Cantonese slow-cooked soup in China

**DOI:** 10.1186/s13002-018-0206-y

**Published:** 2018-01-15

**Authors:** Yujing Liu, Qi Liu, Ping Li, Deke Xing, Huagang Hu, Lin Li, Xuechen Hu, Chunlin Long

**Affiliations:** 1 School of Agricultural Equipment and Engineering, Jiangsu University, Zhenjiang, Jiangsu 212013 China; 2 College of Natural Resources and Environment, South China Agricultural University, Guangzhou, Guangdong 510642 China; 3 Institute of Basic Theory, China Academy of Traditional Chinese Medicine, Beijing, 100700 China; 4 Kunming Institute of Botany, Chinese Academy of Sciences, Kunming, Yunnan 650201 China; 5 College of Life and Environmental Sciences, Minzu University of China, Beijing, 100081 China

**Keywords:** Cantonese slow-cooked soup, Ethnomedicine, Botanical industry, Food therapy, Cultural significance indices

## Abstract

**Background:**

*Lǎo huǒ liàng tāng* (*Cantonese slow-cooked soup*, CSCS) is popular in Guangdong, China, and is consumed by Cantonese people worldwide as a delicious appetizer. Because CSCS serves as an important part of family healthcare, medicinal plants and plant-derived products are major components of CSCS. However, a collated record of the diverse plant species and an ethnobotanical investigation of CSCS is lacking. Because of globalization along with a renewed interest in botanical and food therapy, CSCS has attracted a growing attention in soup by industries, scientists, and consumers. This study represents the first attempt to document the plant species used for CSCS in Guangdong, China, and the associated ethnomedical function of plants, including their local names, part(s) used, flavors, nature, preparation before cooking, habitats, and conservation status.

**Methods:**

In 2014–2017, participatory approaches, open-ended conversations, and semi-structured interviews were conducted with 63 local people and 48 soup restaurant owners (111 interviews) to better understand the biocultural context of CSCS, emphasizing ethnobotanical uses of plants in Guangdong Province, China. Product samples and voucher specimens were collected for taxonomic identification. Mention Index (QI), frequency of use index (FUI), and economic index (EI) were adopted to evaluate the significance of each plant in the food supply.

**Results:**

A total of 97 plant species belonging to 46 families and 90 genera were recorded as having been used in CSCS in the study area. Recorded menus consisted of one or several plant species, with each one used for different purposes. They were classified into 11 functions, with clearing heat being the most common medicinal function. Of the 97 species, 19 grew only in the wild, 8 species were both wild and cultivated, and 70 species were cultivated. Roots and fruits were the most commonly used plant parts in the preparation of CSCS. According to the national evaluation criteria, six of these species are listed on “China’s red list” including two endangered, two critically endangered, one near-threatened, and one vulnerable species. The QI, FUI, and EI of the 97 species in the study varied between 0.09 and 1, 0.23 and 9.95, and 0.45 and 6.58, respectively.

**Conclusions:**

As an important part of Cantonese culture, CSCS has been popularized as a local cuisine with a healthcare function. CSCS also reflects the plant species richness and cultural diversity of Guangdong Province. Future research on the safety and efficacy of CSCS as well as on ecological and cultural conservation efforts is needed for the sustainable growth of China’s botanical and medicinal plant industry.

## Background

In China, many communities have developed their own specific local type of soup, such as *Simmer Soup* in Hunan and Hubei provinces, *Hot and Sour Soup* in Sichuan province, *Mutton Soup* in Shandong province, and *Cantonese slow-cooked soup* (CSCS) in Guangdong Province. Among these soups, CSCS has the greatest number of varieties, and in general, it is well known locally and in foreign countries. As the name implies, CSCS is made with different kinds of ingredients from time to time and is cooked in a covered pot; the pot is allowed to simmer slowly at a low boil on a very low flame for an extended time. CSCS is a relatively low-fat, highly nutritious, and easily absorbed soup, used as a type of delicious appetizer, and has long been a form of traditional food therapy used by Cantonese people.

For a long time, CSCS and cooling herbal teas have epitomized Guangzhou food and drink culture [1]. The origin of CSCS can be traced back to 3500 years ago when it was used as an early form of Chinese herbal medicine [2]. Why? The heat and humidity of Guangdong inevitably penetrate the human body, making people feel very uncomfortable. Because Guangdong features a rich level of biodiversity, Chinese medicinal herbs are available for the Cantonese people to reduce a person’s internal body heat or mitigate the humidity, but pure Chinese herbal tea is very bitter. Medicinal effects without this bitterness were desired. How was this problem solved? Clever Cantonese people added the medicinal herbs, such as ginger (*Zingiber officinale* Rosc.), which is used as an antinauseant [3], *Lophatherum gracile* Brongn., used to cure mouth and tongue sores [4], *Zea mays* L., used to induce diuresis [5], and the seeds of *Euryale ferox* Salisb. ex Konig & Sims, which are used to cure kidney problems, to delicious soups [6].

Cantonese people have brought CSCS into many places where they live, such as Hong Kong, Macao, Taiwan, and other places in Southeast Asia as well as to the Chinatowns of different cities worldwide. CSCS provides a competitive advantage for immigrant Cantonese who markets this product in many places. However, many kinds of CSCS exist, so how does one select the right soup? Because many people lack an awareness of the use of traditional Chinese medicine (TCM) in support of human health, the development of CSCS has become disorganized. In addition, as food security has improved in recent years, international attention has been drawn to food therapy and food safety. As a result, the various types of CSCS need to be analyzed, so that the soup materials can be categorized according to their functions, part(s) used, preparation methods used before decoction and their nature (classified as hot, warm, cool, cold or neutral), and flavor. If these soup materials are classified and used correctly, the opportunity to develop Chinese medicine and expand food variety will emerge. In addition, it is imperative that steps are taken to preserve the heritage we have in TCM along with developing and protecting the nature of CSCS.

## Methods

### Study area

The coastal province of Guangdong is bounded by five southern Chinese provinces along with Hong Kong and Macao. Guangdong Province has a unique style with various dialects, customs, traditions, and historical culture. Guangdong covers an area of 179,800 km^2^ and has 56 ethnic minorities with the Lingnan culture being generally representative. The total population of Han nationality is 102 million in 2013, accounted for 97.46% of Guangdong Province; the population of Zhuang, Yao, Tujia, Miao, and Dong nationality accounts for 86% of the total ethnic minorities’ population in Guangdong. The tropical and subtropical climates have a rich flora that thrives on a variety of geological features.

**Fig. 1:**
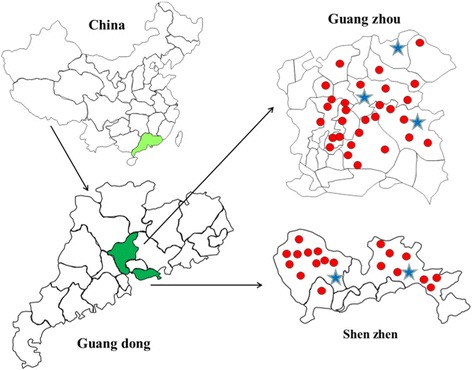
Location of the five villages and 48 restaurants within Guangdong Province in southern China selected as study sites (blue star: village; red dot: restaurant)

While Guangzhou serves as the capital of Guangdong Province, Shenzhen labels itself as an “emerging migrant city” (Fig. 1). Five villages (Mi Gang, Shi Hu, Luo Tang, Long Gang, and Bao An) and 48 restaurants in Guangzhou and Shenzhen were selected as the study sites. The criteria for selecting study sites, including soup chain stores and delivery outlets, were that the sites had a rich variety of CSCS materials so that the soup-drinking culture should be well preserved.

### Ethnobotanical surveys

Our research findings are based on ethnobotanical surveys carried out from 2014 to 2017 with the objective of investigating, documenting, and interpreting which herbs Cantonese consumed in soup used to cure and prevent diseases and how these people produced and consumed these plants. A total of 63 local women in the five villages and 48 soup restaurant owners, both men and women, at other locations (111 interviews) were selected using participatory approaches, open-ended conversations, and semi-structured interviews (Fig. 1) [7]. The restaurant owners, all of whom had lived almost all of their lives in Guangdong, the local female residents over 40 years old, and could cook CSCS were invited to participate as informants; they all readily accepted the invitation to be interviewed. The study was carried out following the International Society of Ethnobiology Code of Ethics; all participants were informed of our intent prior to the start of the interviews. Prior to conducting interviews, we bought or took photos of plant materials used in restaurants in order to conduct a cross-validation of plant identifications in the village; in addition, informants were presented with freshly pressed or gathered plant materials, so the species identifications could be confirmed. During all interviews, the interviewees were asked to speak freely about herb materials to allow us to acquire a list of those species used in making CSCS. In addition, when the interviewees permitted it, samples were collected with the help of local guides. Interviewees were given the option to answer the following questions about each plant species: (1) Have you ever used this plant for making CSCS or not? (2) How often do you eat it? (3) Did you sell or buy this plant? (4) Where do you gather this plant? (5) What plant part do you use of this plant? (6) What is the function of this plant in CSCS? (7) How do you prepare this plant for decoction? (8) How do you obtain plants to cure and prevent diseases in your daily life? (9) Which plants have you used during your lifetime to make CSCS, etc.? Finally, group discussions with key informants were organized separately from the 63 interviews in the five villages. Notes and photos were taken to record the relevant information provided by the informants; digital voice recorders and cameras were used to record the plants and activities of informants as they gathered plants in the field. We collected three specimens of each wild plant. Plants cultivated in home gardens were not used as voucher specimens. For those gathered from the wild and then planted in kitchen gardens, we also gathered the same species of plants from the wild. Voucher specimens of all wild plants available during field investigations were deposited in the Ethnobotany Lab of Minzu University of China. Plant identification was based on the Flora of China, and a review of specimens at PE, the herbarium of the Institute of Botany, Chinese Academy of Sciences, Beijing, China.

### Statistical analysis

Mention Index (QI), frequency of use index (FUI), and Economic Index (EI) were adopted to develop and evaluate a cultural importance index for each plant species [8]. We use questions 1, 2, and 3 above to calculate the QI, FUI, and EI for each species where QI = number of mentions/number of informants. For FUI and EI, the final value of each plant is the average of all informant responses. For the details of the calculation method see Table 1.

**Table 1:** Categorization of answers and values used for the cultural significance indices

Index	Answer	Value
QI	Not mentioned	0
	Mentioned	1
FUI	Never	0
	Less than once a month	2.5
	Once a month	5
	2–3 times a month	7.5
	4 or more a month	10
EI	He/she does not sell or buy it	0
	He/she sells or buys it occasionally at low prices	3.33
	He/she sells or buys it regularly	6.67
	He/she sells or buys it at high prices	10

Table 2 lists the ethnobotanical information for each plant, including scientific name, Chinese name, Cantonese name, function, part(s) used, flavor, nature, preparation before decoction, habitat, conservation need, QI, FUI, and EI based on those defined by the Chinese Pharmacopoeia (National Pharmacopoeia Committee, 2010) and our ethnobotanical surveys. We analyzed the relationship between plant nature, flavor, and function with Apriori and Excel [9]; Apriori is a frequently used item set algorithm that is used for mining association rules. Weka 3.7 software was used to run the association rules program.

**Table 2:** Inventory of plants traditionally used for making *Cantonese slow-cooked soup* in Guangdong, China (species are listed alphabetically)

Scientific name	Chinese name	Chinese character	Cantonese name	Function	Part(s) used	Flavor	Nature	Preparation before decoction	Habitat	Conservation need	QI	FUI	EI	Voucher number
*Adenophora stricta* Miq.	Sha Shen	沙参	Saa sam	Nourish *yin*, stop cough	Root	Sweet, pungent	Warm	Dry	Wild		0.28	0.7	1.53	GD107
*Alisma plantago-aquatica* Linn.	Ze Xie	泽泻	Zaak Se	Clear heat	Stem	Sweet	Cold	Dry	Wild		0.23	0.61	1.2	GD22
*Amomum villosum* Lour.	Sha Ren	砂仁	Saa jan	Tonify *qi*	Fruit	Pungent	Warm	Dry	Cultivated		0.37	0.92	2.34	GD44
*Angelica sinensis* (Oliv.) Diels	Dang Gui	当归	Dong gwai	Replenish blood	Root	Bitter, pungent	Warm	Dry	Cultivated		0.41	1.01	2.34	GD99
*Arachishypogaea* Linn.	Hua Sheng	落花生	Faa sang	Tonify *qi*	Seed	Sweet	Neutral	Dry	Cultivated		0.95	8.58	5.38	GD34
*Armeniaca vulgaris* Lam.	Xing	杏	Hang	Stop cough	Fruit	Sour, sweet	Warm	Fresh	Cultivated		0.73	2	3.72	GD19
*Astragalus membranaceus* (Fisch.) Bunge	Huang Qi	黄耆	Wong kei	Tonify *qi*, tonify *yang*	Root	Sweet	Warm	Dry	Cultivated		0.52	1.69	3.18	GD56
*Atractylodes macrocephala* Koidz.	Bai Zhu	白术	Baak seot	Tonify *qi*	Root	Bitter, sweet	Warm	Dry	Cultivated	VU	0.25	1.01	1.41	GD78
*Benincasa hispida* (Thunb.) Cogn.	Dong Gua	冬瓜	Dung gwaa	Clear heat, stop cough	Fruit	Sweet	Cool	Fresh	Cultivated		0.68	4.08	3.39	GD60
*Brassica pekinensis* (Lour.) Rupr.	Bai Cai	白菜	Baak coi	Digestion, promote dampness	Leaf	Sweet	Neutral	Fresh	Cultivated		0.97	6.78	3.97	GD120
*Carthamus tinctorius* L.	Hong Hua	红花	Hung faa	Promote circulation, tonify *qi*	Flower	Pungent	Warm	Dry	Wild (C)		0.23	0.7	1.5	GD86
*Castanea mollissima* Bl.	Li Zi	栗	Leot zi	Tonify *qi*, promote circulation	Seed	Sweet	Warm	Fresh	Cultivated		0.41	1.17	1.62	GD11
*Chaenomeles sinensis* (Thouin) Koehne	Mu Gua	木瓜	Muk gwaa	Digestion	Fruit	Sour	Warm	Dry	Cultivated		0.44	1.53	2.13	GD200
*Cistanche deserticola* Ma	Rou Cong Rong	肉苁蓉	Juk cung jung	Tonify *yang*	Stem	Sweet, salty	Warm	Dry	Cultivated	CR	0.17	0.43	0.45	GD118
*Citrus limon* (L.) Burm. f.	Ning Meng	柠檬	Ning mung	Clear heat, stop cough	Fruit	Sour, sweet	Neutral	Fresh or dry	Cultivated		0.6	4.53	4.03	GD105
*Citrus reticulata* Blanco	Gan Ju	柑橘	Gam gat	Tonify *qi*	Fruit	Bitter, pungent	Warm	Dry	Cultivated		0.66	5.02	2.88	GD43
*Cocos nucifera* L.	Ye Zi	椰子	Je zi	Tonify *qi*	Fruit	Sweet	Warm	Fresh	Cultivated		0.59	2.73	3.06	GD9
*Codonopsis pilosula* (Franch.) Nannf.	Dang Shen	党参	Dong sam	Tonify *qi*, replenish blood	Root	Sweet	Neutral	Dry	Cultivated		0.34	0.99	2.28	GD6
*Coix lacryma-jobi* L.	Yi Yi	薏苡	Ji ji	Promote dampness, clear heat	Seed	Sweet	Cool	Dry	Cultivated		0.6	3.42	3.82	GD81
*Colocasia esculenta* (L). Schott	Yu	芋	Wu	Tonify *qi*	Bulb	Sweet, pungent	Neutral	Dry	Cultivated		0.87	6.46	4.33	GD67
*Cornus officinalis* Sieb. et Zucc.	Shan Zhu Yu	山茱萸	Saan zyu jyu	Astringents	Fruit	Sour	Warm	Dry	Cultivated		0.39	1.42	2.22	GD87
*Crataegus pinnatifida* Bge.	Shan Zha	山楂	Saan zaa	Digestion	Fruit	Sour, sweet	Warm	Dry	Cultivated		0.74	3.24	3.18	GD54
*Cuscuta chinensis* Lam.	Tu Si Zi	菟丝子	Tou si zi	Tonify *yang*	Seed	Sweet	Warm	Dry	Wild		0.38	1.1	1.53	GD214
*Davallia mariesii* Moore ex Bak.	Gu Sui Bu	骨碎补	Gwat seoi bou	Tonify *yang*	Root	Bitter	Warm	Dry	Wild		0.28	0.7	1.35	GD224
*Dendranthema morifolium* (Ramat.) Tzvel.	Ju Hua	菊花	Guk faa	Clear heat	Flower	Sweet, bitter	Cold	Dry	Wild (cultivated)		0.71	3.06	4.45	GD207
*Dendrobium officinale* Kimura et Migo	Tie Pi Shi Hu	铁皮石斛	Tit pei sek huk	Nourish *yin*, clear heat	Stem	Sweet, salty	Cold	Fresh or dry	Cultivated	CR	0.17	0.43	1.2	GD234
*Dendrobium wilsonii* Rolfe	Guang Dong Shi Hu	广东石斛	Gwong dung sek huk	Nourish *yin*, clear heat	Stem	Sweet	Cold	Fresh or dry	Cultivated	EN	0.09	0.23	0.6	GD244
*Dioscorea esculenta* (Lour.) Burkill	Gan Shu	甘薯	Syu jyu	Tonify *qi*	Root	Sweet	Neutral	Dry	Cultivated		0.47	1.58	2.34	GD177
*Dumasia hirsute* Craib	Ying Mao Shan Hei Dou	硬毛山黑豆	Ngaang mou saan hak dau	Clear heat	Seed	Sweet	Neutral	Fresh	Cultivated		0.53	2.25	2.76	GD109
*Durio zibethinus* Murr.	Liu Lian	榴莲	Lau lin	Nourish *yin*	Fruit	Sweet, pungent	Hot	Fresh	Cultivated		0.25	1.33	1.32	GD21
*Ephedra sinica* Stapf	Cao Ma Huang	草麻黄	Cou maa wong	Promote dampness	Stem	Pungent, bitter	Warm	Dry	Wild		0.19	0.68	0.84	GD117
*Eriobotrya japonica* (Thunb.) Lindl.	Pi Pa	枇杷	Pei paa	Stop cough	Leaf	Bitter	Cold	Dry	Cultivated		0.4	1.31	1.86	GD123
*Eucommia ulmoides* Oliver	Du Zhong	杜仲	Dou zung	Tonify *yang*	Bark	Sweet	Warm	Dry	Wild	NT	0.27	0.77	1.47	GD134
*Euryale ferox* Salisb. ex Konig & Sims	Qian Shi	芡实	Him sat	Astringents	Seed	Sweet, Sour	Neutral	Dry	Cultivated		0.26	1.1	1.65	GD122
*Ficus carica* Linn.	Wu Hua Guo	无花果	Mou faa gwo	Stop cough	Fruit	Sweet	Neutral	Fresh or dry	Cultivated		0.43	1.71	2.49	GD144
*Ficus hirta* Vahl	Cu Ye Rong	粗叶榕	Cou jip jung	Tonify *qi*	Root	Sweet	Warm	Dry	Cultivated		0.25	0.99	0.96	GD199
*Flemingia philippinensis* Merr. et Rolfe	Qian Jin Ba	千斤拔	Cin gan but	Tonify *qi*, promote circulation	Root	Sweet	Neutral	Dry	Cultivated		0.55	2.55	2.91	GD119
*Fritillaria cirrhosa* D. Don	Chuan Bei Mu	川贝母	Cyun bui mou	Stop cough, promote dampness	Bulb	Bitter, sweet	Cold	Dry	Cultivated		0.48	2.39	2.61	GD156
*Ginkgo biloba* L*.*	Yin Xing	银杏	Ngan hang	Stop cough, astringents	Seed	Sweet, bitter, sour	Neutral	Dry	W	EN	0.67	3.81	3.36	GD178
*Glycine max* (Linn.) Merr.	Da Dou	大豆	Daai dau	Clear heat, promote circulation	Seed	Sweet	Neutral	Fresh	Cultivated		0.77	5.25	3.57	GD160
*Hordeum vulgare* L.	Da Mai	大麦	Daai mak	Digestion	Sprout	Sweet	Neutral	Raw or stir-baked form	Cultivated		0.46	2.52	2.28	GD112
*Houttuynia cordata* Thunb	Ji Cai	蕺菜	Jyuu sing cou	Clear heat	Whole plant	Pungent	Cold	Fresh	Wild (cultivated)		0.88	6.67	3.81	GD186
*Hylocereus undatus* (Haw.) Britt. et Rose	Liang Tian Chi	量天尺	Loeng tin cek	Clear heat, stop cough	Flower	Sweet	Cool	Fresh	Wild (cultivated)		0.24	1.04	1.26	GD111
*Ilex pubescens* Hook. et Arn.	Mao Dong Qing	毛冬青	Mou dung cing	Clear heat, promote circulation	Root	Bitter	Cold	Dry	Wild		0.35	1.91	1.74	GD210
*Imperata cylindrica* (L.) Beauv.	Bai Mao	白茅	Baak maau	Clear heat	Root	Sweet	Cold	Dry	Wild		0.51	2.91	2.64	GD218
*Isatis tinctoria* L.	Ou Zhou Song Lan	欧洲菘蓝	Sung Laam	Clear heat	Root	Bitter	Cold	Dry	Wild (cultivated)		0.87	6.67	3.66	GD205
*Jasminum sambac* (L.) Ait.	Mo Li Hua	茉莉花	Mut lei faa	Clear heat	Flower	Pungent, sweet	Warm	Dry	Cultivated		0.71	4.21	3.63	GD243
*Juglans regia* L.	Hu Tao	胡桃	Wu tou	Soothe the nerves and brain	Seed	Sweet	Warm	Dry	Cultivated		0.77	3.81	3.81	GD226
*Juncus bufonius* L.	Xiao Deng Xin Cao	小灯心草	Dang sam cou	Clear heat, promote dampness	Whole plant	Sweet	Cold	Fresh or dry	Wild		0.8	4.03	3.36	GD281
*Lablab purpureus* (Linn.) Sweet	Bian Dou	扁豆	Bin dau	Tonify *yang*	Seed	Sweet	Neutral	Dry	Cultivated		0.11	0.61	0.63	GD267
*Leonurus japonicus* Houtt.	Yi Mu Cao	益母草	Jik mou cou	Promote circulation	Leaf	Bitter, pungent	Cold	Fresh or dry	Wild		0.77	3.11	4.18	GD287
*Ligusticum chuanxiong* Hort.	Chuan Xiong	川芎	Cyun hung	Promote circulation	Root	Pungent	Warm	Fresh or dry	Cultivated		0.37	1.19	2.07	GD254
*Lilium brownie* F. E. Brown ex Miellez	Ye Bai He	野百合	Baak hap	Nourish *yin*, stop cough, soothe the nerves and brain	Leaf	Sweet	Cold	Fresh or dry	Cultivated		0.36	2.18	2.13	GD241
*Lilium lancifolium* Thunb.	Juan Dan	卷丹	Gyun daan baak hap	Nourish *yin*, soothe the nerves and brain	Leaf	Sweet	Cold	Fresh or dry	Cultivated		0.64	3.51	3.57	GD146
*Litchi chinensis* Sonn.	Li Zhi	荔枝	Lai zi	Tonify *qi*, replenish blood, soothe the nerves and brain	Fruit	Sweet, sour	Warm	Fresh	Cultivated		0.77	4.05	4.24	GD165
*Lophatherum gracile* Brongn.	Dan Zhu Ye	淡竹叶	Daam zuk jip	Clear heat, promote dampness	Whole plant	Sweet	Cold	Fresh or dry	Wild		0.78	5.27	4.21	GD110
*Luffa acutangula* (L.) Roem.	Guang Dong Si Gua	广东丝瓜	Si gwaa	Clear heat	Fruit	Sweet	Cool	Fresh	Cultivated		0.88	7.18	4.9	GD119
*Lycium chinense* Mill.	Gou Qi	枸杞	Geoi gei	Nourish *yin*	Fruit	Bitter	Cold	Dry	Cultivated		0.91	7.93	6.58	GD66
*Lycopersicon esculentum* Mill.	Fan Qie	番茄	Faan ke	Digestion	Fruit	Sweet, sour	Cold	Fresh	Cultivated		1	9.95	4.06	GD53
*Magnolia officinalis* Rehd. et Wils.	Hou Pu	厚朴	Hau buk	Clear heat	Flower	Bitter, pungent	Warm	Dry	Cultivated		0.61	3.42	3.12	GD45
*Malus pumila* Mill.	Ping Guo	苹果	Ping gwo	Tonify *qi*, replenish blood	Fruit	Sweet	Cool	Fresh	Cultivated		0.43	2.12	2.61	GD90
*Mentha haplocalyx* Briq.	Bo He	薄荷	Bok ho	Clear heat	Whole plant	Pungent	Cool	Dry or fresh	Cultivated		0.59	4.32	3.06	GD88
*Momordica charantia* L.	Ku Gua	苦瓜	Fu gwaa	Clear heat	Fruit	Bitter	Cold	Fresh	Cultivated		0.53	4.12	2.79	GD142
*Nelumbo nucifera* Gaertn.	Lian	莲	lin	Soothe the nerves and brain	Seed and flower	Sweet, sour	Neutral	Seed: dry/flower: fresh	Cultivated		0.6	3.6	3.33	GD168
*Olea europaea* L.	Mu Xi Lan	木犀榄	Muk sai laam	Clear heat	Fruit	Sweet, sour	Neutral	Fresh	Cultivated		0.35	1.82	0.57	GD175
*Ophiopogon japonicas* (Linn. f.) Ker-Gawl.	Mai Dong	麦冬	Mak dung	Nourish *yin*, stop cough	Root	Sweet, Bitter	Cold	Dry	Wild		0.7	4.26	1.11	GD169
*Oryza sativa* L.	Dao	稻	dou	Nourish *yin*, astringents	Root	Sweet	Neutral	Dry	Cultivated		0.26	1.19	1.08	GD184
*Osmunda japonica* Thunb.	Zi Qi	紫萁	Gun zung	Clear heat	Root	Bitter	Cool	Dry	Cultivated		0.31	1.15	1.65	GD143
*Panax ginseng* C. A. Mey.	Ren Shen	人参	Jan sam	Tonify *qi*, soothe the nerves and brain	Root	Sweet, bitter	Neutral	Dry	Cultivated		0.8	2.91	5.23	GD132
Panax notoginseng (Burkill) F. H. Chen ex C. H. Chow	San Qi/Tian Qi	三七	Saam cat	Promote circulation	Root	Sweet, bitter	Warm	Dry	Cultivated		0.79	3.33	4.96	GD187
*Panax quinquefolius* Linn.	Xi Yang Shen	西洋参	Sai joeng sam	Tonify *qi*, nourish *yin*	Root	Sweet, bitter	Cool	Dry	Cultivated		0.23	0.59	1.53	GD1
*Pinus koraiensis* Sieb. et Zucc.	Hong Song	红松	Hung sung	Nourish *yin*	Seed	Sweet	Warm	Dry	Cultivated		0.58	2.23	3.91	GD91
*Polygonatum cyrtonema* Hua	Duo Hua Huang Jing	多花黄精	Wong zing	Nourish *yin*, tonify *qi*	Root	Sweet	Neutral	Dry	Cultivated		0.53	1.91	2.73	GD58
*Polygonatum odoratum* (Mill.) Druce	Yu Zhu	玉竹	Juk zuk	Nourish yin	Root	Sweet	Cold	Dry	Cultivated		0.58	2.12	0.87	GD65
*Prunella vulgaris* L.	Xia Ku Cao	夏枯草	Haa fu cou	Clear heat	Leaf	Pungent, bitter	Cold	Dry	Wild		0.88	5.83	4.48	GD229
*Pseudostellaria heterophylla* (Miq.) Pax	Hai Er Shen	孩儿参	Taai zi sam	Tonify *qi*	Root	Sweet, bitter	Neutral	Dry	Wild (cultivated)		0.26	0.83	1.65	GD300
*Psoralea corylifolia* Linn.	Bu Gu Zhi	补骨脂	Bou gwat zi	Tonify *yang*, tonify *qi*	Fruit	Pungent, bitter	Warm	Dry	Cultivated		0.22	0.61	1.17	GD209
*Pueraria lobate* (Willd.) Ohwi	Ge Gen	葛	Fan got	Tonify *yang*, astringents	Root	Sweet, pungent	Cool	Dry	Wild		0.32	1.17	1.77	GD273
*Pyrus pyrifolia* (Burm. f.) Nakai	Sha Li	沙梨	Syut lei	Clear heat, stop cough	Fruit	Sweet, sour	Cool	Fresh	Cultivated		0.92	4.64	5.35	GD181
*Quisqualis indica* L.	Shi Jun Zi	使君子	Sai gwan zi	Digestion	Fruit	Sweet	Warm	Dry	Wild (cultivated)		0.26	0.68	1.35	GD315
*Ranunculus ternatus* Thunb.	Mao Zhua Cao	猫爪草	Maau zaau cou	Stop cough	Root	Sweet, pungent	Warm	Dry	Wild		0.19	0.47	0.78	GD320
*Raphanus sativus* L.	Hu Luo Bo	萝卜	Wu lo baak	Digestion	Root	Sweet	Neutral	Fresh	Cultivated		1	9.71	1.71	GD331
*Rehmannia glutinosa* (Gaetn.) Libosch. ex Fisch. et Mey.	Di Huang	地黄	Dei wong	Nourish yin, replenish blood, tonify *qi*	Root	Sweet	Warm	Dry	Wild (cultivated)		0.5	1.78	2.52	GD18
*Rosa laevigata* Michx.	Jin Ying Zi	金樱子	Gam jing zi	Astringents	Fruit	Sour, sweet	Neutral	Dry	Wild		0.62	2	3.3	GD347
*Rosa rugosa* Thunb.	Mei Gui	玫瑰	Mui gwai	Tonify *qi*	Flower	Sweet, bitter	Warm	Dry	Cultivated		1	3.83	4.99	GD10
*Saccharum sinense* Roxb.	Zhu Zhe	竹蔗	Zuk ze	Clear heat, digestion	Juice	Sweet	Neutral	Fresh	Cultivated		1	4.14	4.68	GD121
*Salvia miltiorrhiza* Bunge	Dan Shen	丹参	Daan sam	Promote circulation	Root	Bitter	Cold	Dry	Cultivated		0.34	1.35	2.4	GD316
*Scrophularia ningpoensis* Hemsl.	Xuan Shen	玄参	Duk gok gam	Clear heat, nourish *yin*	Root	Sweet, bitter, salty	Cold	Dry	Cultivated		0.26	0.83	1.74	GD326
*Siraitia grosvenorii* (Swingle) C. Jeffrey ex Lu et Z. Y. Zhang	Luo Han Guo	罗汉果	Lo hon gwo	Stop cough	Fruit	Sweet	Cool	Dry	Cultivated		1	7.66	6.07	GD333
*Stellaria nipponica* Ohwi	Bai HuaFan Lv	多花繁缕	Baak faa se sit cou	Clear heat, promote dampness	Whole plant	Sweet	Cool	Dry	Wild		0.44	2.7	2.22	GD3
*Striga asiatica* (L.) O. Kuntze	Du Jiao Jin	独脚金	Duk gok gam	Clear heat, digestion	Whole plant	Sweet	Cool	Dry	Wild		0.42	2.43	2.34	GD336
*Triticum aestivum* L.	Pu Tong Xiao Mai	普通小麦	Pou tung siu mak	Astringents, tonify *qi*, clear heat	Fruit	Sweet	Cool	Dry	Cultivated		0.35	1.31	1.74	GD312
*Vigna radiata* (Linn.) Wilczek	Lv Dou	绿豆	Luk dau	Clear heat	Seed	Sweet	Cool	Dry	Cultivated		1	8.49	5.83	GD228
*Vigna umbellate* (Thunb.) Ohwi et Ohashi	Chi Xiao Dou	赤小豆	Cik siu dau	Promote dampness	Seed	Sweet, sour	Neutral	Dry	Cultivated		1	7.7	4.63	GD171
*Vigna unguiculata* (Linn.) Walp.	Jiang Dou	豇豆	Gong dau	Digestion	Seed	Sweet	Neutral	Fresh or dry	Cultivated		1	7.34	4.96	GD180
*Zea mays* L.	Yu Shu Shu	玉蜀黍	Juk mai	Promote dampness	Seed	Sweet	Neutral	Fresh	Cultivated		0.87	5.56	4.48	GD50
*Ziziphus jujuba* Mill.	Zao	枣	Mou ci zou	Tonify *qi*	Fruit	Sweet	Warm	Dry	Cultivated		1	8.99	6.07	GD342

## Results and discussion

### Diversity of plants used in CSCS

**Fig. 2:**
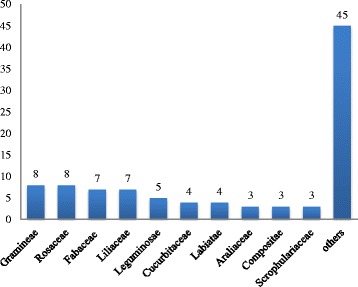
Most frequently mentioned plant families for families where *f* > 3, where *f* is the number of species in a family; for families where *f* < 3, these were summarized as “others”

**Fig. 3:**
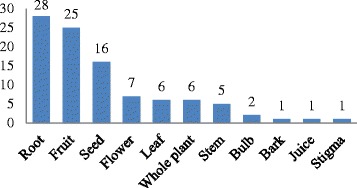
Plant parts used in *Cantonese slow-cooked soup*

Our ethnobotanical surveys documented 113 kinds of plants or plant parts, as defined below, used as ingredients in CSCS (Table 4), including ingredients from 97 species in 90 genera and 46 families (Table 2). In terms of the number of species, the eight species found in each caused the Gramineae and Rosaceae to rank first, followed by seven species each in the Fabaceae and Liliaceae (Fig. 2). The ingredients used in CSCS refer not only to whole herbaceous plants but also to the leaf, bark, root, seed, fruit, stem, bulb, juice, stigma, and flower. Root and fruit were used most commonly. Among these 97 species, 28 and 26 species were collected for the harvesting of roots and fruits, respectively (Fig. 3). Local people also prefer to preserve plants by drying for later use as food materials.

We checked the status of the plant species used in CSCS by following the evaluation criteria established by the International Union for Conservation of Nature (Table 2). Six species are listed on the “China red list”; *Dendrobium officinale* Kimura et Migo and *Cistanche deserticola* Ma are CR (critically endangered), *Dendrobium wilsonii* Rolfe is listed as EN (endangered), and *Atractylodes macrocephala* Koidz. is VU (vulnerable). The remaining 91 species are in the “least concern” category. For the six endangered or vulnerable wild species, reasonable cultivation protocols and ex situ conservation methods need to be established as soon as possible.

The QI of the 97 species in the present study varied between 9 and 100%. The QIs of 46 species were ranked at 0–50% (~ 47.4%) and were 51–99% for 42 species (~ 43.3%); only nine species had a QI of 100% (~ 9.3%). The nine species mentioned above are familiar to almost everyone and include *Lycopersicon esculentum* Mill., *Vigna umbellata* (Thunb.) Ohwi et Ohashi, *Saccharum sinense* Roxb., *Vigna unguiculata* (Linn.) Walp., *Rosa rugosa* Thunb., *Vigna radiata* (Linn.) Wilczek, *Raphanus sativus* L., *Siraitia grosvenorii* (Swingle) C. Jeffrey ex Lu et Z. Y. Zhang, and *Ziziphus jujuba* Mill.

The FUI varied between 0.23 and 9.95. Nineteen species (~ 19.6%) were used more than once a month (FUI > 5). Table 2 clearly shows that the most frequently mentioned species were also the most commonly used, with the exceptions of *R. rugosa* (FUI = 3.83) and *S. sinense* (FUI = 4.14). Fifty-one species were used only occasionally in some years (FUI < 2.5); they are relatively somewhat difficult to obtain either by collection or through commerce.

The EI varied between 0.45 and 6.58. Also, 89 species had an appreciable economic importance (EI > 1). *Lycium chinense* Mill. had the highest EI value (EI = 6.58); *L. chinense* is very significant because most people like to add it to CSCS to flavor the soup and nourish the body.

### Function and five elements of plants

**Fig. 4:**
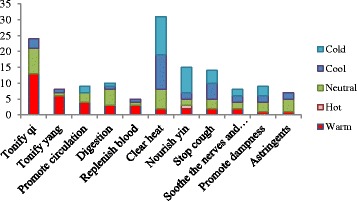
The relationship between five natures (cold, cool, neutral, warm, and hot) and function of plants in CSCS

Chinese people attached great importance to the therapeutic role of food during the early stages of the development of Chinese medicine [10]. CSCS has the concomitant function of serving as both food and medicine based on past experience and the theory of TCM. In the present study, the medicinal functions of CSCS can be classified into 11 categories (Table 2 and Fig. 4). In TCM, “*qi*” is considered to be a natural energy and the central underlying principle of life. Symptoms of various illnesses are believed to be the product of deficiencies or imbalances in the *qi* of the organs of the body [11]. If a *qi* deficiency exists in the spleen, a person will be tired and experience a loss of appetite. If a *qi* deficiency occurs in the lung, a person will experience shortness of breath and cough, have pale skin color, and sweat spontaneously. The Cantonese often relieve these types of imbalances by adjusting the circulation of *qi* using food therapy. During our field surveys, we found 24 species involved in tonifying a person’s *qi* (Fig. 4). In addition, some kinds of CSCS have significant effects in promoting digestion, dampness, and circulation as well as in tonifying a person’s *yang*; these soups will have an astringent, soothing effect on the nerves and brain while replenishing the blood. Chinese philosophy considers *yin* and *yang* to be the two complementing principles of life; *yin* has the female characteristics of earth, cold, and darkness, and *yang* has the male characteristics of heaven, heat, and light. Any one person has both *yin* and *yang*, and these characteristics need to be balanced to maintain good health.

Although the precepts of Chinese food therapy are neither systematic nor identical in different times and places, some basic concepts have been isolated. The Chinese Pharmacopoeia (2010) classifies herbs as having five natures and five flavors based on the five elements theory, which determines their usage. The five natures (hot, warm, cool, cold, and neutral) are summarized mainly from the body’s response after Chinese herbs are consumed [12]. In addition, herbs are classified into five flavors by their efficacy, using their true taste [12] including sweet, bitter, sour, pungent, and salty [13]. The five elements theory is similar with the concept of organoleptic characteristics introduced by Nina Etkin [14], how people use taste to select food or medicine; the taste of plants can define the curing properties or specific diseases of food or medicine, for example, sour tastes are useful for curing fever and nausea. In this study, each herb was linked with the theory of the five elements (Table 3). The natures of these 97 species range from cold (22 species), cool (16), neutral (27), warm (31), and hot (only *Durio zibethinus* Murr.). Because Guangdong is located in the East Asia monsoon region, it experiences a hot and humid climate. Practitioners of TCM hold that inner heat will accumulate in summer, and this causes many types of illness. However, many people prefer to select cool and cold herbs to clear heat from the body. In our study, the most common function of herbs related to their nature was clearing heat, with 31 plant species having this function. Also, a small amount of a hot herb was often used in CSCS, which is consistent with the ethnobotanical survey conducted here.

**Table 3:** The relationship between five plant natures and five plant flavor

Nature	Sweet	Bitter	Sour	Salty	Pungent
Neutral	25	3	7	0	1
Warm	21	8	5	1	11
Cold	14	11	1	2	3
Cool	14	2	1	0	2
Hot	1	0	0	0	1

We analyzed the relationship between plant nature and function. Thirty-eight species having a cool or cold nature, among which 23 species are mainly used to clear heat, account for 60.5%. In addition, we can see that the warm herbs are mainly used to tonify *qi* and *yang*, which accounts for 61.3% of all herbs analyzed (Fig. 4). Aside from tonifying *qi* and clearing heat, neutral herbs are mainly used to aid digestion and as astringents. However, no definite corresponding relationship was found to exist between nature and function.

**Fig. 5:**
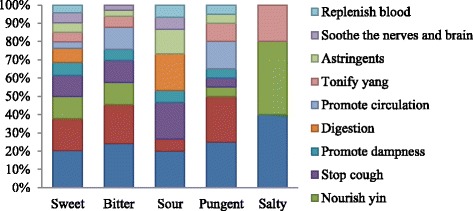
The relationship between plant flavor and function

In TCM, an herb with a sour taste would be assumed to be astringent; an herb with a bitter taste would be useful to eliminate dampness; pungent substances are thought to induce sweat; sweetness is supplementing, harmonizing, and moistening; and saltiness can soften hard masses [13]. In this study, 75, 26, 14, 18, and 3 species were classified as sweet, bitter, sour, pungent, and salty, respectively. We tried to find the corresponding relationship between flavor and function. Here, we indicated that sweet, bitter, and pungent herbs can be used as astringents and not just sour herbs. In addition, the main functions of sour herbs are clearing heat, stopping cough, and helping digestion. The main functions of sweet herbs are clearing heat, tonifying *qi*, nourishing *yin*, and stopping a cough. The main functions of bitter herbs are clearing heat, tonifying *qi*, nourishing *yin*, stopping a cough, and promoting circulation. The main functions of pungent herbs are clearing heat and tonifying *qi* (Fig. 5). Generally, clearing heat is the main function of all herbs. It seems that there is no obvious connection between flavor and function. In fact, substances may also have more than one flavor. For example, *Angelica sinensis* (Oliv.) Diels is sweet and pungent, *Lycopersicon esculentum* Mill. is sweet and sour, and *Scrophularia ningpoensis* Hemsl. is sweet, bitter, and salty. In addition, each herb has its unique nature. Chinese herbal nature is an important part of TCM theory; a single characteristic (a nature, flavor, element, function, etc.) or two such characteristics cannot reveal the internal law of a particular herb systematically. Also, the 97 species discussed here cannot fully reveal the internal law; additional species will need to be analyzed. The relationship between each of the five elements of an herb and its function needs to be studied comprehensively, with the discussion not only confined to CSCS materials. In addition, we should combine the flavor and nature of an herb to explain the complicated relationship between the five elements and function and not separate flavor from nature.

Modern scientific research has been undertaken on the five natures since 1960, mainly in China and Japan, with a focus on pharmacodynamic and substantial foundational research [12]. So far, no definitive compatibility of the five elements and function has been found for Chinese medicinal herbs. The experience from ethnobotanical research related to CSCS will supply some materials for studying the relationship between the five elements and function; this will help to match ingredients with different symptoms.

### How to choose a type of soup?

Cantonese people usually match ingredients based on the symptoms, medicinal effect, seasonal changes, physical quality, job, age, and gender of a patient to help people keep physically fit and to prevent and cure diseases.

1. Soup choice depends on the symptoms: Herbs comprise most of the ingredients in CSCS. Chinese medicine emphasizes matching the remedy to the case [12], so the Cantonese will choose different soups for patients with different symptoms. For example, a mix of *Ziziphus jujuba* Mill. and *Angelica sinensis* (Oliv.) Diels or *Leonurus japonicus* Houtt. was used to regulate menstruation; a mix of *Ligusticum chuanxiong* Hort. and *Dumasia hirsuta* Craib was used to stop coughing and replenish *qi*; see Table 2 for details.
2. Soup choice depends on the season: The Cantonese choose different soups in different seasons. In spring or summer, the Cantonese tend to choose plants to nourish the liver, such as *L. chinense* and *Cuscuta chinensis* Lam., or to nourish the *yin*, such as *S. ningpoensis*, *D. wilsonii*, and *Polygonatum odoratum* (Mill.) Druce. In autumn or winter, the Cantonese tend to choose plants to moisten the lungs, such as *Lilium brownii* F. E. Brown ex Miellez, *Polygonatum cyrtonema* Hua, *Pinus koraiensis* Sieb. et Zucc., and *Panax ginseng* C. A. Mey., or to tonify the kidneys, such as *Rehmannia glutinosa* (Gaetn.) Libosch. ex Fisch. et Mey., *Davallia mariesii* Moore ex Bak., and *Eucommia ulmoides* Oliver.
3. Soup choice depends on physical quality: TCM contains nine kinds of physical qualities known as moderation, *qi* deficiency, *yang* deficiency, phlegm-dampness, blood stasis, *qi* stagnation, *yin* deficiency, damp-heat, and allergic qualities [15]. In our field surveys, menu nos. 77 and 100 are suitable for the *qi* deficiency group; menu nos. 1, 5, 75, 96, 97, and 99 are suitable for the *yang* deficiency group; menu nos. 2 and 101 are suitable for the phlegm-dampness group; menu nos. 15, 73, 102, and 103 are suitable for the blood stasis group; and nos. 104 and 105 are suitable for the *qi* stagnation group (Table 4). Epidemiological studies have shown 14–50% of people with hypertension have a *yin* deficiency. Shen et al. indicated that Chinese food therapy can restore the constitution of the body with a *yin*-*yang* imbalance and may be beneficial in controlling blood pressure in hypertensive patients [16]. *Benincasa hispida* (Thunb.) Cogn., *V. radiata*, and *Glycine max* (Linn.) Merr. are good for nourishing the *yin*.
4. Soup choice depends on job category: Night workers, such as taxi drivers, easily suffer from fatigue and anorexia; therefore, they should choose *Crataegus pinnatifida* Bge. and *Citrus reticulata* Blanco to increase their appetites. Workers who use computers should choose *R. sativus*, *Dendranthema morifolium* (Ramat.) Tzvel., and *L. chinense* to improve vision.
5. Soup choice depends on age and gender: Middle-aged women may have symptoms of endocrine dyscrasia, metabolic disorders, anemia, and osteoporosis caused by hormone problems; for them, the Cantonese would boil *A. sinensis*, *R. glutinosa*, *Z. jujuba*, and *R. sativus* to nourish the *yin* and tonify the blood. Middle-aged men may be given *Psoralea corylifolia* Linn. and *E. ulmoides* to tonify the *yang*. The metabolism of an elderly person may gradually weaken; thus, elderly people should use *Z. jujuba* and *Dioscorea fordii* Prain et Burkill to invigorate the spleen and stomach as well as to tonify the *qi* and the blood.
  CSCS can help to keep a person in good health; however, not everyone will want to eat it because of some taboos related to their diets. Herbs not only have nutrients but also numerous chemical components, some of which are known to have biological actions; however, others may have a potential to influence human physiology and pathophysiology, but this area of research remains unexplored [10]. *Codonopsis pilosula* (Franch.) Nannf. can tonify the *qi*, replenish the blood [17], and has antitumor activity [18, 19]. *Astragalus membranaceus* (Fisch.) Bunge can tonify the *qi* and the *yang* [20, 21]. *Panax quinquefolius* Linn. possesses certain effects on tonifying the *qi* and nourishing the *yin* [22] and is active against human breast cancer [23]. *Polygonatum cyrtonema* Hua can tonify the *qi*, nourish the *yin* [24], and has anti-HIV properties [25]. *Eucommia ulmoides* Oliver can tonify the *yang* [26, 27] and improve the human immune system [28]. In this survey, the Cantonese told us that pregnant women should not eat much *Armeniaca vulgaris* Lam., *Coix lacryma-jobi* L., and *Hordeum vulgare* L. They told us that people who are deficient in cold of the spleen and stomach should not eat much *V. radiata. Panax ginseng* C. A. Mey. and *Panax quinquefolius* Linn. cannot be eaten with *R. sativus* and *C. pinnatifida*. People who suffer from superfluity syndrome or warm syndrome cannot have *C. pilosula*. Patients with acute illnesses cannot have *A. membranaceus*. The Cantonese also told us that people with frequent diarrhea should not eat *P. cyrtonema*, and those with kidney ailments should not eat *E. ulmoides.*

**Table 4:** Menus documented in this study

Menu no.	Plant ingredients (Latin name)	Other ingredients (English name)
1	*Codonopsis pilosula*, *Dioscorea fordii*, *Zingiber officinalis*	Quail, pork
2	*Ficus hirta*, *Flemingia philippinensis*	Chicken
3	*Dumasia hirsuta Craib*	Crucian, chicken
4	*Cornus officinalis*, *Euryale ferox*	Pork
5	*Dioscorea fordii*	Crucian, pork
6	*Dioscorea fordii*, *Euryale ferox*, *Lycium chinense*, *Adenophora stricta*, *Polygonatum odoratum*, *Ziziphusjujuba*, *Zingiber officinalis*	Squab, pork
7	*Striga asiatica*	Pork
8	*Hordeum vulgare*	Duck kidney
9	*Pseudostellaria heterophylla*, *Ficus carica*, *Ziziphus jujuba*, *Zingiber officinalis*	Pork
10	*Amomum villosum*	Pork tripe, chicken, *Hericium erinaceus*
11	*Amomum villosum*	Crucian
12	*Castanea mollissim*, *Dioscorea fordii*, *Ziziphus jujuba*, *Zingiber officinalis*	Trotters
13	*Brassica pekinensis*, *Euryale ferox*, *Coix lacryma-jobi*, *Ziziphus jujuba*, *Citrus reticulata*, *Zingiber officinalis*	Duck, tofu
14	*Pueraria lobata*	Dace
15	*Vigna umbellata*, *Stellaria nipponica*	
16	*Triticum aestivum*, *Codonopsis pilosula*, *Dioscorea fordii*, *Zingiber officinalis*	Pork, beef
17	*Artemisia scoparia*	Crucian
18	*Rosa laevigata*, *Alisma plantago-aquatica*	Pork
19	*Raphanus sativus*, *Zingiber officinalis*	Duck
20	*Chaenomeles sinensis*, *Zingiber officinalis*	Duck, pork
21	*Adenophora stricta*, *Dioscorea fordii*, *Polygonatum odoratum*, *Zingiber officinalis*	Goose, pork
22	*Dumasia hirsuta*, *Citrus reticulata*, *Zingiber officinalis*	Carp, pork
23	*Lilium lancifolium*, *Citrus reticulata*, *Zingiber officinalis*	Crucian, pork
24	*Armeniaca vulgaris*	Crocodile
25	*Eriobotrya japonica* (leaves)	Fish
26	*Chaenomeles sinensis*, *Zingiber officinalis*	Cuttlefish, pork
27	*Astragalus membranaceus*, *Oryza sativa var. glutinosa* (root)	Fish
28	*Vigna unguiculata*, *Zingiber officinalis*	Fish
29	*Oryza sativa var. glutinosa* (root), *Pseudostellaria heterophylla*	Loach
30	*Polygonatum cyrtonema*, *Zingiber officinalis*	Oyster, chicken
31	*Polygonatum odoratum*, *Adenophora stricta*, *Coix lacryma-jobi*, *Zingiber officinalis*	Pork, tendon
32	*Armeniaca vulgaris*, *Pyrus pyrifolia*, *Ephedra sinica*, *Ziziphus jujuba*	Pork
33	*Malus pumila*, *Ephedra sinica*, *Ziziphus jujuba*	Pork, tremella
34	*Olea europaea*	Conch, pork
35	*Glycine max*, *Sauropus spatulifolius (leave)*	Crucian
36	*Panax quinquefolius*	Pork
37	*Ginkgo biloba* (fruit), *Nelumbo nucifera*, *Zingiber officinalis*	Chicken
38	*Glycine max*, *Momordica charantia*	Pork ribs
39	*Raphanus sativus*, *Ziziphus jujuba*, *Citrus reticulata*, *Zingiber officinalis*	Pork ribs
40	*Nelumbo nucifera*, *Luffa acutangula*, *Zingiber officinalis*	Chicken
41	*Panax quinquefolius*, *Dioscorea fordii*, *Ziziphus jujuba*, *Zingiber officinalis*	Squab
42	*Vigna radiata*, *Lilium lancifolium*, *Panax quinquefolius*	Squab
43	*Arachis hypogaea*, *Astragalus membranaceus*, *Ziziphus jujuba*	Beef
44	*Nelumbo nucifera*, *Litchi chinensis*, *Zingiber officinalis*	Duck
45	*Dendrobium wilsonii*, *Dioscorea fordii*, *Lycium chinense*, *Citrusӱeticulate*	Pork
46	*Dendrobium wilsonii*, *Pyrus pyrifolia*	Duck
47	*Dendrobium officinale*, *Ophiopogon japonicus*, *Ziziphus jujuba*	Pork
48	*Dendrobium wilsonii*, *Lycium chinense*	Pork liver
49	*Angelica sinensis*, *Ziziphus jujuba*	
50	*Leonurus japonicas*, *Ziziphus jujuba*	
51	*Pinus koraiensis*, *Panax quinquefolius*	Chicken or pork
52	*Pinus koraiensis*	Pork
53	*Arachis hypogaea, Citrus reticulata*	Pork
54	*Olea europaea*, *Castanea mollissima*, *Raphanus sativus*	Quail, pork
55	*Hylocereus undatus* (flower), *Imperata cylindrica*, *Armeniaca vulgaris*, *Ziziphus jujuba*	Pork lung
56	*Dioscorea fordii*, *Ziziphus jujube*, *Zingiber officinalis*	Pork
57	*Laminaria japonica*, *Vigna unguiculata*, *Panax notoginseng*	A: scorpion, pork; B: squab
58	*Vigna radiata*, *Lilium lancifolium*, *Dendrobium officinale*, *Panax quinquefolius*	
59	*Vigna radiate*, *Momordica charantia*	Pork
60	*Lycium chinense*, *Zingiber officinalis*, *Allium fistulosum*	Beef
61	*Quisqualis indica*	Pork
62	*Dendrobium officinale*, *Polygonatum odoratum*, *Adenophora stricta*	Pork
63	*Glycine max*, *Cocos nucifera*, *Ficus carica*, *Zingiber officinalis*	Chicken
64	*Ranunculus ternatus*, *Zingiber officinalis*	Pork
65	*Eucommia ulmoides*, *Psoralea corylifolia*, *Zingiber officinalis*	Pork ribs
66	*Dioscorea fordii*, *Zingiber officinalis*	Fish, pork
67	*Lablab purpureus*, *Arachis hypogaea*, *Zea mays*, *Zingiber officinalis*	Fish, pork
68	*Ficus carica*, *Arachis hypogaea*, *Zingiber officinalis*	Pork, tripe
69	*Durio zibethinus*, *Zingiber officinalis*	Crucian
70	*Dioscorea fordii*, *Euryale ferox*, *Nelumbo nucifera*	Hippocampus, pork
71	*Ilex pubescens*, *Lycium chinense*, *Cuscuta chinensis*, *Rehmannia glutinosa*, *Zingiber officinalis*	Pork
72	*Lycopersicon esculentum*, *Daucus carota subsp. sativus*, *Zingiber officinalis*, *Allium fistulosum*	Pork
73	*Ligusticum* chuanxiong, *Dumasia hirsuta*	Pork
74	*Ranunculus ternatus*, *Prunella vulgaris*, *Glycine max*	Pork
75	*Dendrobium officinale*, *Panax quinquefolius*, *Dioscorea fordii*	Chicken or pork
76	*Saccharum sinense*, *Raphanus sativus*, *Citrus reticulata*, *Zingiber officinalis*	Pork
77	*Codonopsis pilosula*, *Lilium lancifolium*, *Zingiber officinalis*	Squab
78	*Euryale ferox*, *Zingiber officinalis*	Chitterlings, scallops
79	*Nelumbo nucifera*, *Zingiber officinalis*	Carp
80	*Fritillaria cirrhosa*, *Eriobotrya japonica* (leaves)	Fish, pork
81	*Salvia miltiorrhiza*	Chicken
82	*Nelumbo nucifera*, *Castanea mollissima*, *Zingiber officinalis*	Pork kidney
83	*Armeniaca vulgaris*, *Pyrus pyrifolia*, *Lilium lancifolium*	Goose
84	*Dumasia hirsuta*, *Triticum aestivum*, *Rehmannia glutinosa*, *Zingiber officinalis*, *Citrus reticulata*	Oyster, pork
85	*Euryale ferox*, *Juglans regia*, *Dioscorea fordii*, *Zingiber officinalis*	Pork kidney
86	*Astragalus membranaceus*, *Ziziphus jujube*, *Zingiber officinalis*	Eel, pork kidney
87	*Ficus carica*, *Ziziphus jujube*, *Zingiber officinalis*	Chicken
88	*Colocasia esculenta*, *Zingiber officinalis*	Pork, scallops
89	*Allium fistulosum*, *Zingiber officinalis*	Chicken, mushroom
90	*Eucommia ulmoides*, *Cistanche deserticola*	Pork
91	*Nelumbo nucifera*, *Vigna umbellata*, *Zingiber officinalis*, *Ziziphus jujuba*	Squid, pigeon
92	*Mentha haplocalyx*, *Magnolia officinalis*	Pork
93	*Atractylodes macrocephala*	Crucian
94	*Houttuynia cordata*, *Siraitia grosvenorii*	Pork lung
95	*Pyrus pyrifolia*, *Armeniaca vulgaris*	Jellyfish
96	*Lycium chinense*, *Ziziphus jujube*	Chicken
97	*Angelica sinensis*, *Zingiber officinale*	Mutton
98	*Angelica sinensis*, *Rehmannia glutinosa*	Squab
99	*Dioscorea fordii*, *Raphanus sativus*, *Ziziphus jujube*, *Lycium chinense*	Chicken
100	*Panax ginseng*, *Ziziphus jujube*, *Lycium chinense*	Silkie
101	*Coix lacryma-jobi*, *Lablab purpureus*, *Citrus reticulate*, *Ziziphus jujube*	Squab
102	*Crataegus pinnatifida*, *Raphanus sativus*	Pork feet
103	*Rosa rugosa*, *Carthamus tinctorius*, *Angelica sinensis*	Pork
104	*Dendranthema morifolium*, *Jasminum sambac*	Chicken liver, tremella
105	*Citrus limon*, *Ziziphus jujube*, *Lycium chinense*	Chicken
106	*Lophatherum gracile, Juncus bufonius*, *Ophiopogon japonicus*	
107	*Rehmannia glutinosa*, *Scrophularia ningpoensis*	Pork
108	*Panax notoginseng*, *Ziziphus jujube*	Frog
109	*Rehmannia glutinosa, Carthamus tinctorius, Angelica sinensis*	Silkie
110	*Davallia mariesii*	Pork
111	*Isatis tinctoria*, *Osmunda japonica*	Pork
112	*Benincasa hispida*	Crucian
113	*Dioscorea fordii*, *Zea mays*	

## Conclusions

The number of groups of people with less than robust health continues to increase. In the long course of development of CSCSs, the quintessence of TCM has been adopted. Different soups have different functions. CSCS has four dimensions: social, functional, cultural, and economic. As soup materials, traditional knowledge of various plants used in CSCS was documented, including local plant name, function, part(s) used, flavor, nature, preparation before decoction, habitat, and cultural significance indices. Knowledge of these herbs used in food therapy will provide a broad socio-anthropological context related to eating. The relationships among the nature, flavor, and function of herbs seem to be related to each other but are not absolute, which will be a key point of consideration in TCM. In addition, these theories of CSCS will provide the essential basis for the analyses and clinical usage of Chinese herbs.

## Abbreviations

CR: Critically endangered
CSCS: Cantonese slow-cooked soup
EI: Economic Index
EN: Endangered
FUI: Frequency of use index
QI: Mention Index
TCM: Traditional Chinese medicine
VU: Vulnerable

## References

[1] Liu YJ, Ahmed S, Long CL (2013). Ethnobotanical survey of cooling herbal drinks from southern China. J Ethnobiol Ethnomed.

[2] Cai SF. The development and research on nutritional therapy soup and diet of Hong Kong: Guangzhou University of Chinese Medicine; 2009.

[3] Fulder S, Tenne M. Ginger as an anti-nausea remedy in pregnancy the issue of safety. HerbalGram (USA). 1996;1(12):2521-31.

[4] Shao Y, Wu Q, Wen H, Chai C, Shan C, Yue W, Yan S, Xu H (2014). Determination of flavones in lophatherum gracile by liquid chromatography tandem mass spectrometry. Instrum Sci Technol.

[5] Doan DD, Nguyen N, Doan H, Nguyen T, Phan T, Van Dau N, Grabe M, Johansson R, Lindgren G, Stjernström N (1992). Studies on the individual and combined diuretic effects of four Vietnamese traditional herbal remedies (*Zea mays*, *Imperata cylindrica*, *Plantago major* and *Orthosiphon stamineus*). J Ethnopharmacol.

[6] Zhang S, Cheng H, Dong J (2011). Amino-acid and mineral composition of the seeds of *Euryale ferox*. Chem Nat Compd.

[7] Maria S, Maddalena P, Piero B. Plants and traditional knowledge: an ethnobotanical investigation on Monte Ortobene (Nuoro, Sardinia). J Ethnobiol Ethnomed. 2009;5:1-14. 10.1186/1746-4269-5-6 PMC2661884 19208227

[8] Garibayorijel R, Caballero J, Estradatorres A, Cifuentes J (2007). Understanding cultural significance, the edible mushrooms case. J Ethnobiol Ethnomed.

[9] Yu HY, Xu CG (2013). Relationship between nature and other properties of traditional Chinese medicine based on association rule. Chin J Exp Tradit Med Formulae.

[10] Mclean AJ, Wahlqvist ML. Current problems in nutrition pharmacology and toxicology. Herts: John Libbey; 1988.

[11] Ni M. The Yellow Emperor’s classic of medicine. Boulder: Shambhala Publications; 1995.

[12] Liao H, Banbury LK, Leach DN (2008). Antioxidant activity of 45 Chinese herbs and the relationship with their TCM characteristics. Evid Based Complement Alternat Med.

[13] Ung CY, Li H, Cao ZW, Li YX, Chen YZ (2007). Are herb-pairs of traditional Chinese medicine distinguishable from others? Pattern analysis and artificial intelligence classification study of traditionally defined herbal properties. J Ethnopharmacol.

[14] Thornburg GK (2011). Nina L. Etkin: edible medicines: an ethnopharmacology of food. J Agric Environ Ethics.

[15] Wang Q (2005). Classification and diagnosis basis of nine basic constitutions in Chinese medicine. J Beijing Univ Tradit Chin Med.

[16] Shen C, Pang SMC, Kwong EWY, Cheng Z (2010). The effect of Chinese food therapy on community dwelling Chinese hypertensive patients with yin-deficiency. J Clin Nurs.

[17] Xue-mei M (2009). Advances in studies on *Codonopsis pilosula*. J Anhui Agric Sci.

[18] Xin T, Zhang F, Jiang Q, Chen C, Huang D, Li Y, Shen W, Jin Y, Sui G (2012). The inhibitory effect of a polysaccharide from *Codonopsis pilosula* on tumor growth and metastasis in vitro. Int J Biol Macromol.

[19] Yang C, Gou Y, Chen J, An J, Chen W, Hu F (2013). Structural characterization and antitumor activity of a pectic polysaccharide from *Codonopsis pilosula*. Carbohydr Polym.

[20] Zhang Q, Gao WY, Man SL (2012). Chemical composition and pharmacological activities of *Astragali radix*. China J Chin Mater Med.

[21] Dong TT, Ma XQ, Clarke C, Song ZH, Ji ZN, Lo CK, Tsim KW (2003). Phylogeny of *Astragalus* in China: molecular evidence from the DNA sequences of 5S rRNA spacer, ITS, and 18S rRNA. J Agric Food Chem.

[22] Guo YQ, Wei GL, Zhong QM, Wang DS (2011). Clinical application of *Panax quinquefolius*. Chin J Mod Drug Appl.

[23] Wang C, Aung H, Zhang B, Sun S, Li X, He H, Xie J, He T, Du W, Yuan C (2008). Chemopreventive effects of heat-processed *Panax quinquefolius* root on human breast cancer cells. Anticancer Res.

[24] Chen Y, Sun XS (2010). Pharmacological research grogress in *Polygonatum cyrtonema*. Tradis Chin Drug Res Clin Pharmacol.

[25] Ding JJ, Bao JK, Zhu DY, Zhang Y, Wang DC (2010). Crystal structures of a novel anti-HIV mannose-binding lectin from *Polygonatum cyrtonema* Hua with unique ligand-binding property and super-structure. J Struct Biol.

[26] Xiao L, Zhou RG (2013). The research progresses of *Eucommia ulmoides*’ antihypertensive effect. Chin Med Guide.

[27] Liu N, Shu KX, Liu CS (2002). The domestic and abroad research progresses of *Eucommia ulmoides*. Med J Natl Def Forces Southwest China.

[28] Feng H, Fan J, Song Z, Du X, Chen Y, Wang J, Song G (2016). Characterization and immunoenhancement activities of *Eucommia ulmoides* polysaccharides. Carbohydr Polym.

